# Metropolitan Hospital Sunday Fund

**Published:** 1907-01-19

**Authors:** 


### METROPOLITAN HOSPITAL SUNDAY FUND.

A meeting of the Council of the Metropolitan Hospital Sunday Fund was held at the Mansion House on Thursday, January 10, the Lord Mayor (Sir William Treloar) presiding, for the election of committees and honorary officers for the ensuing year.

The Secretary (Sir Edmund Hay Currie) said he had received a letter from Canon Pycke resigning his position on the Council, on the ground of inability to attend meetings because of advancing age. It is usual to fill clerical vacancies by members of the same denomination, and as a Roman Catholic had resigned he submitted the name of the Rev. Monsignor Howlett, Administrator of the Cathedral at Westminster, who was recommended by Archbishop Bourne. This suggestion was adopted, and the Secretary was directed to write to the Bishop of Southwark to nominate a South London clergyman to take the place of the Rev. R. S. Hassard, resigned.

To fill lay vacancies on the Council the following gentlemen were adopted, subject to their consent: Dr. James Andrews, of Hampstead, Mr. Frank Debenham, Mr. C. E. Layton, Mr. Alexander Millar, and Mr. A. G. Phillips.

The Distribution Committee was re-elected, with Mr. Frank Bevan, Sir Joseph Dimsdale, and Sir Frederick Treves in place of Sir Sydney Waterlow and Mr. George Herring, deceased, and Sir Felix Schuster, who had resigned owing to his appointment to the India Council.

The General Purposes Committee was also re-elected, with the addition of Lord Cheylesmore.

The Secretary then brought forward a suggestion for the appointment of a Finance Committee. The Fund, he said, had never had such a Committee, but now they had large matters to deal with, such, for instance, as Mr. Herring's estate, it was thought desirable to appoint one. He thought five would be a suitable number. This was agreed to without discussion, and the following gentlemen were appointed: Alderman Sir John Bell, Mr. J. H. Buxton, Sir William Church, Mr. John Hampton Hale, and Sir Ernest Tritton.

The hon. secretaries, Sir Richard Martin and Mr. John S. Gilliat, and the hon. auditors, Messrs. W. H. Pannell and Company, were also re-elected, and the proceedings then terminated.

